# Time use and mental health in UK adults during an 11-week COVID-19 lockdown: a panel analysis

**DOI:** 10.1192/bjp.2021.44

**Published:** 2021-10

**Authors:** Feifei Bu, Andrew Steptoe, Hei Wan Mak, Daisy Fancourt

**Affiliations:** Department of Behavioural Science and Health, Institute of Epidemiology & Health Care, University College London, UK

**Keywords:** Time use, depression, anxiety, life satisfaction, COVID-19

## Abstract

**Background:**

There is currently major concern about the impact of the global COVID-19 outbreak on mental health. But it remains unclear how individual behaviours could exacerbate or protect against adverse changes in mental health.

**Aims:**

To examine the associations between specific activities (or time use) and mental health and well-being among people during the COVID-19 pandemic.

**Method:**

Data were from the UCL COVID-19 Social Study, a panel study collecting data weekly during the COVID-19 pandemic. The analytical sample consisted of 55 204 adults living in the UK who were followed up for the 11-week strict lockdown period from 21 March to 31 May 2020. Data were analysed using fixed-effects and Arellano–Bond models.

**Results:**

Changes in time spent on a range of activities were associated with changes in mental health and well-being. After controlling for bidirectionality, behaviours involving outdoor activities such as gardening and exercising predicted subsequent improvements in mental health and well-being, whereas increased time spent following news about COVID-19 predicted declines in mental health and well-being.

**Conclusions:**

These results are relevant to the formulation of guidance for people obliged to spend extended periods in isolation during health emergencies and may help the public to maintain well-being during future lockdowns and pandemics.

A number of studies have demonstrated the negative psychological effects of the coronavirus disease 2019 (COVID-19) pandemic.^1,2^ However, much of the research to date has focused on the mass behaviour of ‘staying at home’ as the catalyst, with little exploration into how specific behaviours within the home might differentially affect mental health. The COVID-19 pandemic has had a profound impact on how people spend their time, such as cessation of work (through unemployment or furloughs), increased childcare responsibilities and home-schooling, and a sharp curtailing of leisure activities, with shopping, day trips, going to entertainment venues, face-to-face social interactions and most activities in public spaces prohibited during ‘lockdowns’.^3^ There is a substantial literature on the relationship between the ways people spend their time and mental health. Certain behaviours have been proposed to exert protective effects on mental health, such as taking up a hobby,^4^ engaging in physical activity^5,6^ and broader leisure activities such as reading, listening to music and volunteering.^7,8^ However, other behaviours may have a negative influence on mental health, such as productive activities (e.g. long working hours)^9^ and sedentary screen time.^10^ This relationship between time use and mental health could be bidirectional, with mental ill health also affecting motivation to engage in activities. Yet to date, there have been few data on the association between daily activities and mental health among people staying at home during the COVID-19 pandemic. Further, it is unclear whether activities that are usually beneficial for mental health have similar psychological benefits during the pandemic. This topic is pivotal as understanding time use will help in formulating healthcare guidelines for individuals continuing to stay at home owing to quarantine, shielding or virus resurgences during the COVID-19 pandemic and in potential future lockdowns and pandemics.

This study involved analyses of longitudinal data from over 50 000 adults captured during the first lockdown due to the COVID-19 pandemic in the UK. It explored the time-varying relationship between a wide range of activities and mental health, including productive activities (e.g. paid work, volunteering, housework), exercising, gardening, reading for pleasure, hobby, communicating with others, following news on COVID-19 and sedentary screen time. We focused on three different aspects of mental health: anxiety, combining negative mood states with physiological hyperarousal; depression, combining negative mood states with anhedonia (loss of pleasure); and life satisfaction, an assessment of how favourable one feels towards one's attitude to life.^11,12^ Therefore, this study sought to disentangle differential associations between time use and multiple aspects of mental health. As these relationships can be complex and are likely bidirectional, this study explored (a) concurrent changes in behaviours and mental health to identify associations over time and (b) whether changes in behaviours temporally predicted changes in mental health, accounting for the possibility of reverse causality by using dynamic panel methods.

## Method

### Participants

Data were drawn from the UCL COVID-19 Social Study, a large panel study of the psychological and social experiences of over 50 000 adults (aged 18+) in the UK during the COVID-19 pandemic. The study commenced on 21 March 2020 and it involves online weekly data collection from participants for the duration of the COVID-19 pandemic in the UK. Although not random, the study has a heterogeneous sample that was recruited using three primary approaches. First, convenience sampling was used, including promoting the study through existing networks and mailing lists (including large databases of adults who had previously consented to be involved in health research across the UK), print and digital media coverage, and social media. Second, more targeted recruitment was undertaken focusing on (a) individuals from a low-income background, (b) individuals with no or few educational qualifications and (c) individuals who were unemployed. Third, the study was promoted via partnerships with third-sector organisations to vulnerable groups, including adults with pre-existing mental illness, older adults and carers. The study was approved by the UCL Research Ethics Committee (12467/005) and all participants gave informed consent. The full study protocol, including details on recruitment, retention and weighting is available at www.covidsocialstudy.org.

In the study presented here, we focused on participants who had at least two repeated measures between 21 March and 31 May 2020, when the UK went into strict lockdown on the 23 March and remained largely in that situation until 1 June (although the lockdown measures started to be eased earlier in different UK nations). This provided us with data from 55 204 participants (total observations: 338 083; observations per person: mean 6.1, range 2–11).

### Measures

Depression was measured using the Patient Health Questionnaire (PHQ-9), a standard instrument for diagnosing depression in primary care.^13^ Unlike the original PHQ-9, this study asked about ‘over the last week’ instead of ‘over the last two weeks’, as data were collected weekly. The questionnaire involves nine items, with four-point responses ranging from ‘not at all’ to ‘nearly every day’. The total score ranges from 0 to 27 and higher overall scores indicate more depressive symptoms.

Anxiety was measured using the Generalized Anxiety Disorder Assessment (GAD-7), a well-validated tool used to screen and diagnose generalised anxiety disorder in clinical practice and research.^14^ These questions were worded as ‘over the last week’ for the same reason as the depression questions. There are seven items with four-point responses ranging from ‘not at all’ to ‘nearly every day’. The total score ranges from 0 to 21, with higher overall scores indicating more symptoms of anxiety.

Life satisfaction was measured by a single question on a scale of 0 to 10: ‘Overall, in the past week, how satisfied have you been with your life?’.^15^

Thirteen measures of time use/activities were considered. These were: (a) working (remotely or outside of the house); (b) volunteering; (c) household chores (e.g. cooking, cleaning, tidying, ironing, online shopping, etc.) or caring for others, including friends, relatives or children; (d) looking after children (e.g. bathing, feeding, doing homework or playing with children); (e) gardening; (f) exercising outside (including going out for a walk or other gentle physical activity, or moderate- or high-intensity activity such as running, cycling or swimming) or inside the home or garden (e.g. doing yoga, weights or indoor exercise); (g) reading for pleasure; (h) engaging in home-based arts or crafts activities (e.g. painting, creative writing, sewing, playing music); engaging in digital arts activities (e.g. streaming a concert, virtual tour of a museum) or doing DIY, woodwork, metal work, model making or similar; (i) communicating with family or friends (including phoning, video talking and communicating via email, WhatsApp, text or other messaging service); (j) following information on COVID-19 (e.g. watching, listening to or reading news, or tweeting, blogging or posting about COVID-19); (k) watching TV, films, Netflix, etc. (*not* for information on COVID-19); (l) listening to the radio or music; and (m) browsing the internet, tweeting, blogging or posting content (*not* for information on COVID-19). Each measure was coded as rarely (<30 min), low (30 min to 2 h) and high (≥3 h), except for low-intensity activities such as volunteering, gardening, exercising, reading and arts/crafts, which were coded as none, low (<30 min) and high (≥30 min). We used a ‘stylised questions’ approach in which participants were asked to focus on a single day and consider how much time they spent on each activity on the list. However, given concerns about the cognitive burden of focusing on a ‘typical’ day (which involves aggregating information from multiple days and averaging), we asked participants to focus just on the last weekday (either the day before or the last day prior to the weekend if participants answered on a Saturday or Sunday). This approach follows aspects of the ‘time diary’ approach, but we chose weekday to remove variation in responses due to whether participants took part on weekends.^16^

### Analysis

Data analyses started by using standard fixed-effects models. Fixed-effects analysis has the advantage of controlling for unobserved individual heterogeneity and therefore eliminating potential biases in the estimates of time-variant variables in panel data. It uses only within-individual variation, which can be used to examine how the change in time use is related to the change in mental health within individuals over time. As individuals are compared with themselves over time, all time-invariant factors (such as gender, age, income, education, area of living) are accounted for automatically, even if unobserved. However, fixed-effects analysis does not address the directionality of an association. Therefore, to explore directionality, we further employed the Arellano–Bond approach, which specifies that the dependent variable depends on its values in previous periods.^17^ This is done by including one or more lags of the outcome variable in a first-difference model:
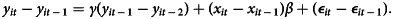
Ordinary least squares regression of the first-difference model produces inconsistent estimates because *y*_*it* − 1_ − *y*_*it* − 2_ and 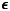
_*it*_ − 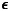
_*it* − 1_ are correlated. To address this problem, the Arellano–Bond model uses *y*_*it* − 2_ and further lags as instruments for*y*_*it* − 1_ − *y*_*it* − 2_. The rationale is that the lagged outcomes are unrelated to the error term in the first difference, 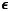
_*it*_ − 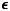
_*it* − 1_, under a testable assumption that 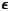
_*it*_ are serially uncorrelated. This assumption is tested using the Arellano–Bond test. Further, we treated the regressors *x*_*it*_ as endogenous (*E*(*x*_*it*_
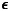
_*is*_) ≠ 0 if *s* ≤ *t*, *E*(*x*_*it*_
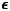
_*is*_) = 0 if *s* > *t*). Therefore, *x*_*it*_ is instrumented by *x*_*it* − 2_, *x*_*it* − 3_ and potentially further lags. The joint validity of instruments is tested by the Sargan–Hansen test.

To account for the non-random nature of the sample, all data were weighted to the proportions of gender, age, ethnicity, education and country of living obtained from the Office for National Statistics.^18^ To address multiple testing, we provided adjusted *P*-values (*q*-values) controlling for the positive false discovery rate. These were generated by using the Stata ‘qqvalue’ package. Moreover, measurement invariance of the latent constructs, namely depression and anxiety, was inspected and tested to ensure meaningful comparisons across time (supplementary Fig. 2, supplementary Box 1 and supplementary Table 1, available at https://dx.doi.org/10.1192/bjp.2021.44). All main analyses were carried out using Stata version 15 for Windows and the Arellano–Bond models were fitted using the user-written command ‘xtabond2’.

## Results

### Descriptive

**Fig. 1:**
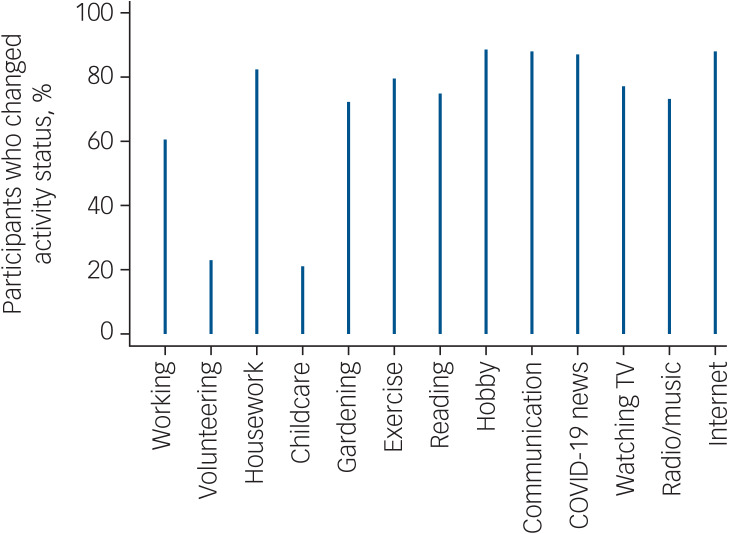
Percentages of participants changing status on the time-use/activity variables across time.

Demographic characteristics of participants are shown in supplementary Table 2. Supplementary Table 3 presents the distribution of the time-use variables at baseline. As shown in Table 1, within-individual variation accounted for about 15% of the overall variation in depression and 16% in anxiety (see also supplementary Fig. 1). Anxiety explained 56% of the variance in depression (*r* = 0.75, *P* < 0.001) and 27% of the variance in life satisfaction (*r* = −0.52, *P* < 0.001), and depression explained 32% of the variance in life satisfaction (*r* = −0.57, *P* < 0.001). There were also substantial changes in the time-use/activity variables (Fig. 1). Over 60% of participants changed status on all activities, except for volunteering (23%) and childcare (21%).

**Table 1:** Summary statistics for depression, anxiety and life satisfaction

	Depression (PHQ-9)		Anxiety (GAD-7)		Life satisfaction	
Variation	Mean	s.d.	Mean	s.d.	Mean	s.d.
Overall	6.09	5.70	4.73	5.11	5.89	2.28
Between individual		5.43		4.84		2.04
Within individual		2.22		2.08		1.10

PHQ-9, nine-item Patient Health Questionnaire; GAD-7, seven-item Generalized Anxiety Disorder Assessment.

### Depression

Increases in time spent working, doing housework, gardening, exercising, reading, engaging in hobbies and listening to the radio/music were all associated with decreases in depressive symptoms (Table 2, Model I-i). The largest decrease in depression was seen for participants who increased their exercise levels to more than 30 min per day, who increased their time gardening to more than 30 min per day, or who increased their work to more than 2 h per day. On the contrary, increasing time spent following COVID-19 news or doing other screen-based activities (either watching TV or internet use/social media) was associated with an increase in depressive symptoms.

**Table 2:** Results from the fixed-effects models on depression, anxiety and life satisfaction

	Model I-i Depression				Model II-i Anxiety				Model III-i Life satisfaction			
	Coefficient	s.e.	*P*	*q*^a^	Coefficient	s.e.	*P*	*q*^a^	Coefficient	s.e.	*P*	*q*^a^
Working 30 min to 2 h (Ref <30 min)	−0.03	0.04	0.458	0.518	0.03	0.04	0.364	0.494	0.01	0.02	0.497	0.562
Working ≥3 h (Ref <30 min)	−**0.27**	**0**.**04**	**0**.**000**	**0**.**000**	0.06	0.04	0.095	0.190	**0**.**11**	**0**.**02**	**0**.**000**	**0**.**000**
Volunteering <30 min (Ref none)	0.00	0.06	0.947	0.958	−0.01	0.05	0.819	0.836	0.00	0.03	0.934	0.934
Volunteering ≥30 min (Ref none)	−0.16	0.10	0.104	0.142	−0.09	0.06	0.148	0.252	**0**.**09**	**0**.**04**	**0**.**028**	**0**.**046**
Housework 30 min to 2 h (Ref <30 min)	−**0**.**11**	**0**.**03**	**0**.**000**	**0**.**000**	**−**0.04	0.03	0.155	0.252	**0**.**05**	**0**.**01**	**0**.**001**	**0**.**002**
Housework ≥3 h (Ref <30 min)	−**0**.**21**	**0**.**05**	**0**.**000**	**0**.**000**	**−**0.04	0.04	0.360	0.494	**0**.**06**	**0**.**02**	**0**.**004**	**0**.**009**
Looking after children 30 min to 2 h (Ref <30 min)	−0.02	0.07	0.744	0.806	0.07	0.07	0.283	0.433	0.04	0.04	0.293	0.381
Looking after children ≥3 h (Ref <30 min)	0.08	0.10	0.435	0.514	0.17	0.09	0.054	0.128	0.05	0.05	0.314	0.389
Gardening <30 min (Ref none)	−**0**.**15**	**0**.**03**	**0**.**000**	**0**.**000**	**−0**.**15**	**0**.**03**	**0**.**000**	**0**.**000**	**0**.**06**	**0**.**02**	**0**.**000**	**0**.**000**
Gardening ≥30 min (Ref none)	−**0**.**30**	**0**.**04**	**0**.**000**	**0**.**000**	**−0**.**24**	**0**.**03**	**0**.**000**	**0**.**000**	**0**.**16**	**0**.**02**	**0**.**000**	**0**.**000**
Exercising <30 min (Ref none)	−**0**.**19**	**0**.**04**	**0**.**000**	**0**.**000**	**−**0.03	0.03	0.437	0.541	**0**.**10**	**0**.**02**	**0**.**000**	**0**.**000**
Exercising ≥30 min (Ref none)	−**0**.**39**	**0**.**04**	**0**.**000**	**0**.**000**	**−0**.**23**	**0**.**03**	**0**.**000**	**0**.**000**	**0**.**22**	**0**.**02**	**0**.**000**	**0**.**000**
Reading <30 min (Ref none)	−0.07	0.03	0.041	0.063	−0.06	0.03	0.048	0.125	0.03	0.02	0.090	0.130
Reading ≥30 min (Ref none)	−**0**.**14**	**0**.**04**	**0**.**001**	**0**.**002**	**−0**.**19**	**0**.**04**	**0**.**000**	**0**.**000**	**0**.**05**	**0**.**02**	**0**.**006**	**0**.**012**
Hobby <30 min (Ref none)	−0.06	0.03	0.052	0.075	−0.01	0.03	0.836	0.836	0.02	0.01	0.117	0.160
Hobby ≥30 min (Ref none)	−**0**.**17**	**0**.**03**	**0**.**000**	**0**.**000**	**−0**.**10**	**0**.**03**	**0**.**000**	**0**.**000**	**0**.**09**	**0**.**01**	**0**.**000**	**0**.**000**
Communication 30 min to 2 h (Ref <30 min)	−0.05	0.03	0.037	0.060	0.04	0.03	0.134	0.249	**0**.**04**	**0**.**01**	**0**.**001**	**0**.**002**
Communication ≥3 h (Ref <30 min)	0.00	0.04	0.958	0.958	**0**.**11**	**0**.**04**	**0**.**004**	**0**.**013**	**0**.**06**	**0**.**02**	**0**.**008**	**0**.**015**
COVID-19 news 30 min to 2 h (Ref <30 min)	**0**.**29**	**0**.**02**	**0**.**000**	**0**.**000**	**0**.**48**	**0**.**02**	**0**.**000**	**0**.**000**	**−0**.**15**	**0**.**01**	**0**.**000**	**0**.**000**
COVID-19 news ≥3 h (Ref <30 min)	**0**.**56**	**0**.**05**	**0**.**000**	**0**.**000**	**0**.**89**	**0**.**04**	**0**.**000**	**0**.**000**	**−0**.**28**	**0**.**02**	**0**.**000**	**0**.**000**
Watching TV 30 min to 2 h (Ref <30 min)	−0.03	0.04	0.423	0.514	−0.03	0.04	0.380	0.494	0.02	0.02	0.332	0.392
Watching TV ≥3 h (Ref <30 min)	**0**.**13**	**0**.**05**	**0**.**016**	**0**.**028**	0.02	0.05	0.638	0.721	−0.04	0.02	0.065	0.099
Listening to radio/music 30 min to 2 h (Ref <30 min)	−**0**.**09**	**0**.**03**	**0**.**003**	**0**.**006**	**−**0.04	0.03	0.089	0.190	**0**.**03**	**0**.**02**	**0**.**027**	**0**.**046**
Listening to radio/music ≥3 h (Ref <30 min)	−**0**.**24**	**0**.**05**	**0**.**000**	**0**.**000**	**−**0.09	0.04	0.031	0.090	**0**.**09**	**0**.**02**	**0**.**000**	**0**.**000**
Internet/social media 30 min to 2 h (Ref <30 min)	0.04	0.03	0.160	0.208	−0.01	0.02	0.775	0.836	−0.01	0.01	0.701	0.759
Internet/social media ≥3 h (Ref <30 min)	**0**.**11**	**0**.**04**	**0**.**015**	**0**.**028**	0.02	0.04	0.519	0.613	0.00	0.02	0.878	0.913
Number of observations	308 182				308 182				308 182			
Number of individuals	54 632				54 632				54 632			

Ref, reference; bold denotes significance at *q* < 0.05.

a. *q*-values are *P*-values controlling for the positive false discovery rate.

When examining the direction of the relationship (Table 3, Model I-ii), increases in gardening, exercising, reading and listening to the radio/music predicted subsequent decreases in depressive symptoms. However, increases in time spent following news on COVID-19 predicted increases in depressive symptoms, as did increases in time spent looking after children or moderate increases in communicating with others via video calls, phone or messaging.

**Table 3:** Results from the Arellano–Bond models on depression, anxiety and life satisfaction

	Model I-ii Depression				Model II-ii Anxiety				Model III-ii Life satisfaction			
	Coefficient	s.e.	*P*	*q*^a^	Coefficient	s.e.	*P*	*q*^a^	Coefficient	s.e.	*P*	*q*^a^
Working 30 min to 2 h (Ref <30 min)	−0.04	0.39	0.920	0.955	−0.02	0.33	0.962	0.995	−0.42	0.18	0.021	0.052
Working ≥3 h (Ref <30 min)	0.23	0.27	0.391	0.573	0.60	0.24	0.012	0.065	−**0.39**	**0**.**13**	**0**.**003**	**0**.**014**
Volunteering <30 min (Ref none)	−0.08	0.56	0.887	0.955	−0.67	0.58	0.244	0.507	0.34	0.35	0.326	0.518
Volunteering ≥30 min (Ref none)	−0.34	0.60	0.570	0.770	−0.08	0.50	0.875	0.995	**0**.**69**	**0**.**26**	**0**.**009**	**0**.**027**
Housework 30 min to 2 h (Ref <30 min)	−0.05	0.31	0.862	0.955	0.03	0.27	0.903	0.995	0.01	0.15	0.929	0.929
Housework ≥3 h (Ref <30 min)	0.02	0.49	0.974	0.974	−0.16	0.44	0.711	0.995	0.07	0.25	0.773	0.835
Looking after children 30 min to 2 h (Ref <30 min)	**1**.**82**	**0**.**69**	**0**.**008**	**0**.**027**	0.88	0.66	0.185	0.432	−0.59	0.36	0.105	0.203
Looking after children ≥3 h (Ref <30 min)	1.88	0.83	0.023	0.056	1.96	0.81	0.015	0.068	−**1**.**29**	**0**.**42**	**0**.**002**	**0**.**014**
Gardening <30 min (Ref none)	−**1**.**37**	**0**.**30**	**0**.**000**	**0**.**000**	**−0**.**95**	**0**.**28**	**0**.**001**	**0**.**007**	**0**.**75**	**0**.**15**	**0**.**000**	**0**.**000**
Gardening ≥30 min (Ref none)	−**1**.**28**	**0**.**29**	**0**.**000**	**0**.**000**	**−**0.51	0.27	0.060	0.231	**1**.**02**	**0**.**15**	**0**.**000**	**0**.**000**
Exercising <30 min (Ref none)	−0.31	0.35	0.374	0.573	0.07	0.31	0.824	0.995	**0**.**41**	**0**.**17**	**0**.**017**	**0**.**046**
Exercising ≥30 min (Ref none)	−**1**.**06**	**0**.**37**	**0**.**004**	**0**.**015**	0.00	0.32	0.995	0.995	**0**.**50**	**0**.**17**	**0**.**003**	**0**.**014**
Reading <30 min (Ref none)	−0.14	0.40	0.730	0.918	−0.03	0.33	0.921	0.995	0.04	0.18	0.844	0.876
Reading ≥30 min (Ref none)	−0.76	0.38	0.046	0.089	−0.51	0.31	0.107	0.321	0.33	0.18	0.064	0.144
Hobby <30 min (Ref none)	0.73	0.33	0.029	0.065	0.47	0.30	0.126	0.340	0.05	0.16	0.749	0.835
Hobby ≥30 min (Ref none)	0.04	0.28	0.891	0.955	−0.18	0.27	0.511	0.812	0.16	0.14	0.256	0.432
Communication 30 min to 2 h (Ref <30 min)	**0**.**71**	**0**.**29**	**0**.**013**	**0**.**035**	0.46	0.27	0.082	0.277	0.20	0.14	0.156	0.281
Communication ≥3 h (Ref <30 min)	0.91	0.45	0.045	0.089	0.29	0.39	0.454	0.766	0.11	0.22	0.619	0.796
COVID-19 news 30 min to 2 h (Ref <30 min)	**0**.**77**	**0**.**19**	**0**.**000**	**0**.**000**	**1**.**26**	**0**.**17**	**0**.**000**	**0**.**000**	**−0**.**25**	**0**.**09**	**0**.**006**	**0**.**020**
COVID-19 news ≥3 h (Ref <30 min)	**0**.**78**	**0**.**31**	**0**.**011**	**0**.**033**	**1**.**38**	**0**.**27**	**0**.**000**	**0**.**000**	**−0**.**42**	**0**.**15**	**0**.**006**	**0**.**020**
Watching TV 30 min to 2 h (Ref <30 min)	0.40	0.48	0.403	0.573	0.12	0.45	0.793	0.995	0.07	0.25	0.768	0.835
Watching TV ≥3 h (Ref <30 min)	0.45	0.54	0.400	0.573	−0.47	0.52	0.362	0.652	−0.11	0.28	0.704	0.835
Listening to radio/music 30 min to 2 h (Ref <30 min)	−**1**.**16**	**0**.**37**	**0**.**002**	**0**.**009**	**−**0.04	0.32	0.911	0.995	−0.13	0.18	0.481	0.684
Listening to radio/music ≥3 h (Ref <30 min)	−**1**.**57**	**0**.**51**	**0**.**002**	**0**.**009**	0.61	0.46	0.192	0.432	0.18	0.25	0.468	0.684
Internet/social media 30 min to 2 h (Ref <30 min)	0.11	0.34	0.748	0.918	−0.07	0.30	0.805	0.995	0.09	0.16	0.560	0.756
Internet/social media ≥3 h (Ref <30 min)	0.87	0.49	0.075	0.135	0.42	0.44	0.346	0.652	−0.38	0.23	0.098	0.203
First lag of the outcome variable	0.10	0.02	0.000	0.000	0.18	0.02	0.000	0.000	0.08	0.01	0.000	0.000
Number of observations	159 470				159 470				159 470			
Number of individuals	42 400				42 400				42 400			

Ref, reference; bold denotes significance at *q* < 0.05.

a. *q*-values are *P*-values controlling for the positive false discovery rate. The serial correlation test showed a negative first-order serial correlation but no second-order correlation, meaning that the model was correctly specified.

### Anxiety

Increases in time spent gardening, exercising, reading and other hobbies were all associated with decreases in anxiety, whereas increasing time spent following COVID-19 news and communicating remotely with family/friends were associated with increases in anxiety (Table 2, Model II-i). The largest decrease in anxiety was seen for participants who increased their time on gardening, exercising or reading to 30 min or more per day.

When looking at the direction of the relationship (Table 3, Model II-ii), increases in gardening predicted a subsequent decrease in symptoms of anxiety. But increasing time spent following news on COVID-19 predicted an increase in anxiety.

### Life satisfaction

Increases in time spent working, volunteering, doing housework, gardening, exercising, reading, engaging in hobbies, communicating remotely with family/friends and listening to the radio/music were all associated with an increase in life satisfaction, whereas increasing time spent following COVID-19 news was associated with a decrease in life satisfaction (Table 2, Model III-i).

When looking at the direction of the relationship (Table 3, Model III-ii), increases in volunteering, gardening and exercising predicted a subsequent increase in life satisfaction. But increasing time spent following news on COVID-19, working and looking after children predicted a decrease in life satisfaction.

### Sensitivity analyses

We carried out sensitivity analyses excluding keyworkers, who might not have been isolated at home in the same way and therefore might have had different patterns of behaviour during lockdown (*n* = 41 728). The results were materially consistent with the main analysis (supplementary Tables S4 and S5 excluding key workers and supplementary Tables 6 and 7 for key workers). Other sensitive analyses controlling for anxiety when modelling depression and *vice versa* (supplementary Table 8) and restricting the sample to people with at least three repeated measures (supplementary Table 9) are also provided.

## Discussion

This is the first study to examine the impact of time use on mental health among people during the COVID-19 pandemic. Time spent on work, housework, gardening, exercising, reading, hobbies, communicating with friends/family and listening to music were all associated with improvements in mental health and well-being, whereas following the news on COVID-19 (even for only half an hour a day) and watching television excessively were associated with declines in mental health and well-being. Although the relationship between time use and behaviours is bidirectional, when exploring the direction of the relationship using lagged models, behaviours involving outdoor activities such as gardening and exercising predicted subsequent improvements in mental health and well-being, whereas time spent watching the news about COVID-19 predicted declines in mental health and well-being.

### Negative associations

Our findings of negative associations between following the news on COVID-19 and mental health echo a cross-sectional study from China showing that social media exposure during the pandemic is associated with depression and anxiety.^19^ The fact that exposure to COVID-19 news is largely screen-based, and the fact that watching high levels of television or high social media engagement unrelated to COVID-19 were also found to be associated with depression, could suggest that this finding is more about the screens than the news specifically.^20^ However, the association with following the news on COVID-19 was independent of these other screen behaviours and was found for even relatively low levels of exposure (30 min to 2 h per day). Further, there have been wider discussions of the negative impact of news during the pandemic, including concerns about the proliferation of misinformation and sensationalised stories on social media^21^ and information overload, whereby the amount of information exceeds people's ability to process it.^22^ It is notable that these associations were found for all measures of mental ill health and well-being and even in lagged models that attempted to remove the effects of reverse causality, suggesting the strength of its relationship with mental health.

### Protective associations

However, other activities were shown to have protective associations with mental health. In particular, outdoor activities such as gardening and exercise were associated with better levels of mental health and well-being across all measures, with many of these results were maintained in lagged models. These results echo many previous studies into the benefits of outdoor activities.^4–6^ Exercise (including gentle activities such as gardening) can affect mental health via physiological mechanisms (such as reducing blood pressure), neuroendocrine mechanisms (such as reducing levels of cortisol involved in the stress response), neuroimmune mechanisms (including reducing levels of inflammation associated with depressive symptoms and increasing the synthesis and release of neurotransmitters and neurotrophic factors associated with neurogenesis and neuroplasticity) and psychological mechanisms (including improving self-esteem, autonomy and mood).^23^ Particularly during lockdown, such activities (which provided opportunities to leave the home) may have helped in providing physical and mental separation from fatiguing or stressful situations at home, offering a change of scenery and providing a feeling of being connected to something larger.^24^

Hobbies such as listening to music, reading and engaging in arts and other projects were also associated with better mental health across all measures. This builds on substantial literature showing the benefits of such activities in reducing depression and anxiety, building a sense of self-worth and self-esteem, fostering self-empowerment and supporting resilience.^7^ The associations presented here show that these activities have remained beneficial to mental health during lockdown. However, these associations were not retained as consistently across lagged models. This suggests that they may be linked more bidirectionally with mental health, with changes in mental health also driving individuals’ motivation to engage with these activities.

### Equivocal associations

There are several other noteworthy findings from these analyses. First, volunteering was associated with higher levels of life satisfaction, including across lagged models that explored the direction of association, but not with other aspects of mental health. Previous studies have demonstrated psychological benefits of volunteering, but our findings suggest that it plays a specific role in supporting evaluative well-being during the pandemic.^8^ Second, both work and housework had some protective associations when looking at parallel changes with mental health over time. However, when looking at lagged models, housework does not appear to be a precursor to changes in mental health, and frequent working was associated with lower life satisfaction, independent of other types of predictor. This echoes research highlighting working from home as a cause of stress for many people during the COVID-19 pandemic.^3^ Similarly, looking after children was not associated with changes in mental health in our main models, but increases to high volumes of childcare were associated with higher levels of depression and lower life satisfaction over time. The fact that there was not an increase in anxiety could suggest that parents did not find such experiences a source of threat, but instead felt lower mood owing to either increased strain or not having the time for personal self-care activities. Communicating with family/friends, on the other hand, had mixed effects in our main models, but when exploring the direction of association, it was in fact associated with higher levels of depression. This could be explained by data from previous studies showing that although face-to-face interactions can decrease loneliness (which is associated with mental ill health, including depression), communication over the telephone (or other digital means) can in certain circumstances increase loneliness, perhaps as it is perceived as a less emotionally rewarding experience.^25^ Finally, depression and anxiety are highly correlated measures: patients with depression are likely to have features of anxiety disorder and *vice versa*. However, our analyses show some differential associations of time use/activities with depression and anxiety. For instance, working and listening to radio/music are unidirectionally associated with depression, but no evidence is found for anxiety. These differences remain to be explored in future studies.

### Strengths and limitations

This study has a number of strengths, including its large sample size, repeated weekly follow-up of the same participant over the 11 weeks of the first UK lockdown and robust statistical approaches being applied. Notably, we had repeated measures using the same validated scales. However, the UCL COVID-19 Social Study did not use a random sample. Nevertheless, the study does have a large sample with wide heterogeneity, including good stratification across all major sociodemographic groups. In addition, analyses were weighted on the basis of population estimates of core demographics, with the weighted data showing good alignment with national population statistics and another large-scale nationally representative social survey. But we cannot rule out the possibility that the study inadvertently attracted individuals experiencing more extreme psychological experiences, with subsequent weighting for demographic factors failing to fully compensate for these differences. This study looked at adults in the UK in general, but it is likely that ‘lockdown’ or ‘stay at home’ orders had different impact on time use for people with different sociodemographic characteristics, for example age and gender. Although our analyses statistically took account of all stable participant characteristics (even if unobserved) by comparing participants with themselves, future studies could examine how the relationship between time use and mental health differs by individuals’ characteristics and backgrounds. We also lack data to see how behaviours during lockdown compared with behaviours prior to the pandemic, so it remains unknown whether changes such as increasing time spent on childcare or leisure activities were unusual for participants and therefore not part of their usual coping strategies for their mental health. Moreover, time spent on each activity was measured as a categorical variable, the categorisation of which was arguably arbitrary. Further studies will benefit from using a continuous measure of time. Finally, although we standardised our questions to the last weekday and used the same response with all participants consistently across lockdown (which is well recognised as an approach in tracking time use, as discussed in the Methods section), it is nevertheless possible that behaviours over the weekends might also have been influencing mental health independent of weekday behaviours. Future studies may want to extend the findings here to explore how behaviours during lockdown are associated with mental health experiences as lockdowns ease.

### Implications

In our exploration of parallel changes in time use and mental health, our finding that many behaviours commonly identified as important for good mental health (e.g. hobbies, listening to music and reading for pleasure) were associated with improved well-being and lower symptoms of mental illness attests to the importance of both encouraging health-promoting behaviours to support mental health and understanding mental health when setting guidelines on healthy behaviours during a pandemic. In exploring the direction of the relationships, our findings that changes in outdoor activities (e.g. exercise and gardening) were strongly associated with subsequent changes in mental health and that increasing exposure to news on COVID-19 was strongly associated with declines in mental health are important in formulating guidance for people likely to experience enforced isolation for months to come (due to quarantine, self-isolation or shielding) and are also key in preparing for future lockdowns and pandemics so that more targeted advice can be given to individuals to help them stay well at home.

## Data Availability

Anonymised data will be made available after the end of the pandemic.

## Supplementary material

For supplementary material accompanying this paper visit https://doi.org/10.1192/bjp.2021.44. click here to view supplementary material
