# Supplementary material for: Time use and mental health in UK adults during an 11-week COVID-19 lockdown: a panel analysis
Source: Br J Psychiatry. 2021 Oct;219(4):551–6. doi: 10.1192/bjp.2021.44 (PMC8481937; doi:10.1192/bjp.2021.44)
Supplement: Supplementary file 1 [file S0007125021000441sup001.pdf]

Supplementary Material

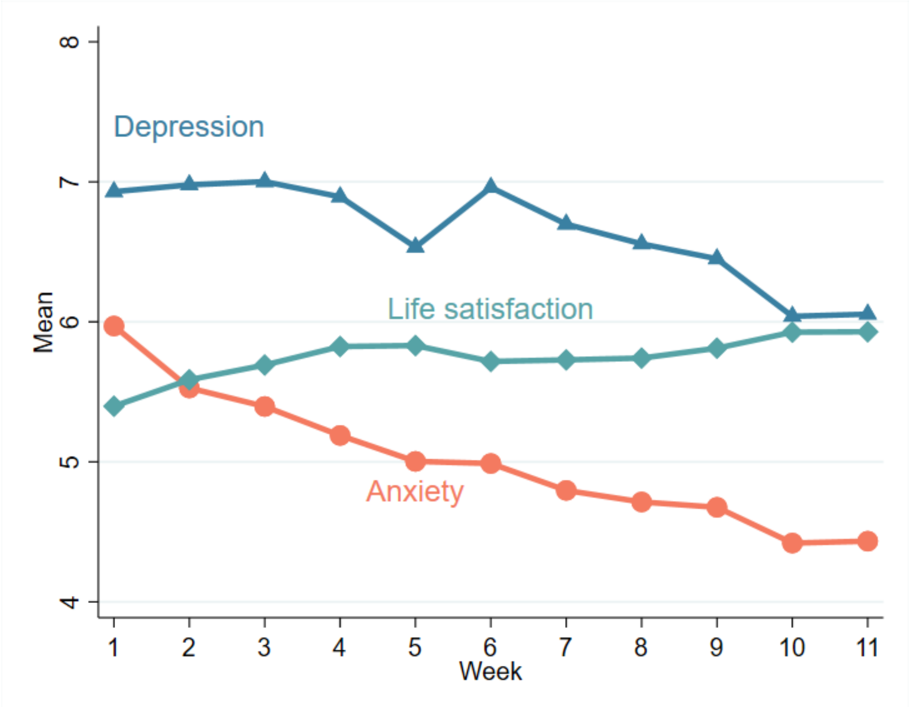

Figure S1. Descriptive changes of depression, anxiety and life satisfaction over time

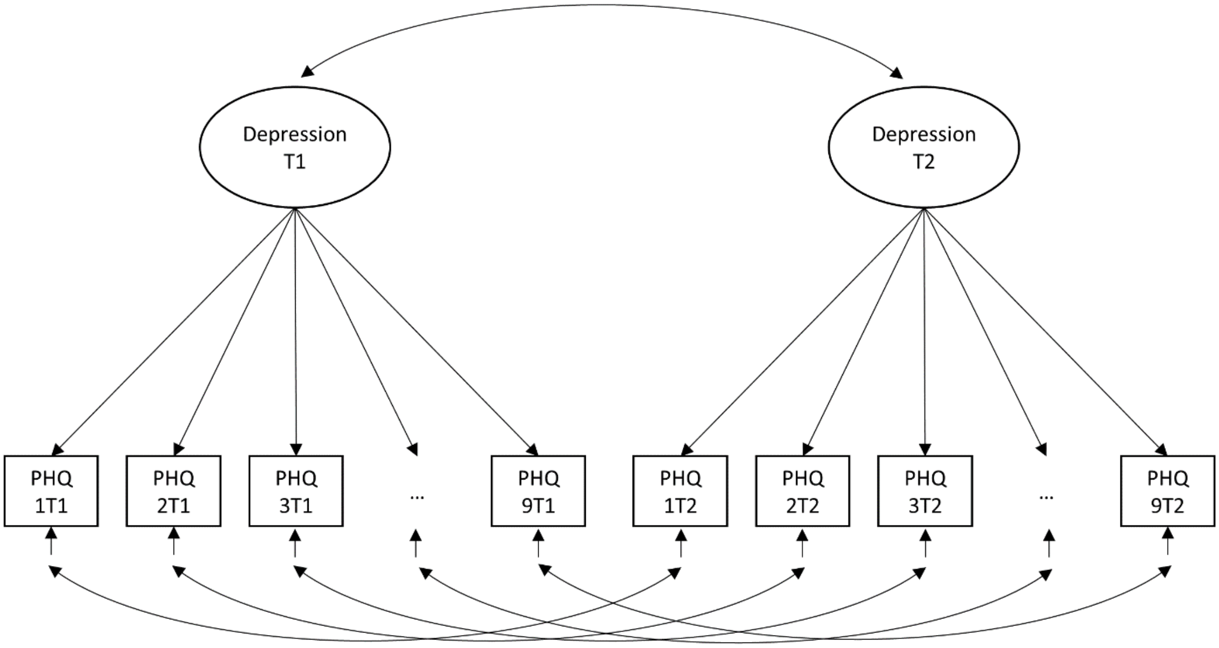

Figure S2. Model specification for testing longitudinal measurement invariance (depression)

	Estimate	S.E.	Est./S.E.	Two-Tailed P-Value
DEP1 BY				
PHQ1T1	1.000	0.000	999.000	999.000
PHQ2T1	1.058	0.020	51.786	0.000
PHQ3T1	1.004	0.032	30.908	0.000
PHQ4T1	1.016	0.026	39.093	0.000
PHQ5T1	0.974	0.029	33.736	0.000
PHQ6T1	0.890	0.026	34.843	0.000
PHQ7T1	0.949	0.030	32.107	0.000
PHQ8T1	0.444	0.033	13.441	0.000
PHQ9T1	0.439	0.028	15.626	0.000
DEP2 BY				
PHQ1T2	1.000	0.000	999.000	999.000
PHQ2T2	1.043	0.014	76.586	0.000
PHQ3T2	1.031	0.023	44.119	0.000
PHQ4T2	1.051	0.021	49.280	0.000
PHQ5T2	0.884	0.026	33.372	0.000
PHQ6T2	0.904	0.023	39.876	0.000
PHQ7T2	0.946	0.026	37.083	0.000
PHQ8T2	0.375	0.022	17.408	0.000
PHQ9T2	0.423	0.024	17.633	0.000
Intercepts				
PHQ1T1	1.773	0.018	98.703	0.000
PHQ2T1	1.779	0.018	96.942	0.000
PHQ3T1	2.001	0.020	100.555	0.000
PHQ4T1	2.131	0.019	112.480	0.000
PHQ5T1	1.808	0.021	85.737	0.000
PHQ6T1	1.512	0.019	79.877	0.000
PHQ7T1	1.711	0.017	102.249	0.000
PHQ8T1	1.218	0.013	93.193	0.000
PHQ9T1	1.177	0.013	92.843	0.000
PHQ1T2	1.762	0.016	108.909	0.000
PHQ2T2	1.736	0.017	102.247	0.000
PHQ3T2	2.006	0.020	101.457	0.000
PHQ4T2	2.029	0.019	108.751	0.000
PHQ5T2	1.735	0.019	91.629	0.000
PHQ6T2	1.527	0.018	83.730	0.000
PHQ7T2	1.644	0.019	85.355	0.000
PHQ8T2	1.169	0.010	115.925	0.000
PHQ9T2	1.185	0.011	104.946	0.000

Box S1. Results from the longitudinal confirmatory factory analysis model (configural invariance model)

Table S1 Comparing model fit indices across longitudinal factor analysis models

Model	RMSEA	CFI	SRMR
Configural invariance	0.025	0.947	0.037
Weak measurement invariance	0.025	0.946	0.039
Strong measurement invariance	0.025	0.943	0.039
Strict measurement invariance	0.028	0.922	0.055

Notes: RMSEA=root mean square error of approximation, CFI=comparative fit index, SRMR=standardized root mean square residual

Table S2. Demographic characteristics (N= 55,204 weighted)

	Variables	Percentages
Age	18-29	18.4%
	30-45	27.3%
	46-59	24.4%
	60+	29.9%
Gender	Women (vs. men)	50.0%
Ethnicity	BAME (vs. white)	12.5%
Education	GCSE or below	32.0%
	A-levels or equivalent	33.5%
	Degree or above	34.5%
Household income	Low (<30k) (vs. high)	47.3%
Area of living	City	34.4%
	Large town	20.7%
	Small town	24.6%
	Village, hamlet or other	20.3%
Key worker role	Key worker	22.5%

Note: all observed and unobserved time-invariant demographic characteristics are automatically accounted for in fixed-effects models but are shown here for descriptive purposes.

Table S3 Distribution of the time use variables at baseline (N=55,204 Weighted)

Variables	Percentages
Working (<30mins)	49.4%
Working (30mins-2hrs)	10.9%
Working (≥3hrs)	39.7%
Volunteering (none)	91.7%
Volunteering (<30mins)	3.6%
Volunteering (≥30mins)	4.7%
Housework (<30mins)	26.2%
Housework (30mins-2hrs)	49.2%
Housework (≥3hrs)	24.6%
Looking after children (<30mins)	80.3%
Looking after children (30mins-2hrs)	8.0%
Looking after children (≥3hrs)	11.7%
Gardening (none)	52.9%
Gardening (<30mins)	18.1%
Gardening (≥30mins)	29.0%
Exercising (none)	21.5%
Exercising (<30mins)	24.9%
Exercising (≥30mins)	53.6%
Reading (none)	44.6%
Reading (<30mins)	21.9%
Reading (≥30mins)	33.5%
Hobby (none)	48.2%
Hobby (<30mins)	18.0%
Hobby (≥30mins)	33.8%
Communication (<30mins)	28.4%
Communication (30mins-2hrs)	45.9%
Communication (≥3hrs)	25.7%
COVID-19 news (<30mins)	33.7%
COVID-19 news (30mins-2hrs)	42.3%
COVID-19 news (≥3hrs)	24.0%
Watching TV (<30mins)	8.2%
Watching TV (30mins-2hrs)	36.3%
Watching TV (≥3hrs)	55.5%
Listening radio/music (<30mins)	27.1%
Listening radio/music (30mins-2hrs)	49.0%
Listening radio/music (≥3hrs)	23.9%
Internet/social media (<30mins)	26.7%
Internet/social media (30mins-2hrs)	44.5%
Internet/social media (≥3hrs)	28.7%

Table S4 Results from the fixed-effects models on depression, anxiety and life satisfaction (excluding key workers)

	Model I-i Depression				Model II-i Anxiety				Model III-i Life satisfaction			
	Coef.	SE	p	q†	Coef.	SE	p	q†	Coef.	SE	p	q†
Working 30mins-2hrs (Ref. <30mins)	0.00	0.05	0.977	0.999	0.04	0.04	0.368	0.581	0.01	0.02	0.598	0.676
Working ≥3hrs (Ref. <30mins)	-0.24	0.05	0.000	0.000	0.09	0.04	0.041	0.107	0.09	0.02	0.000	0.000
Volunteering <30mins (Ref. none)	0.00	0.07	0.999	0.999	-0.06	0.05	0.295	0.548	0.03	0.03	0.278	0.344
Volunteering ≥30mins (Ref. none)	-0.23	0.11	0.046	0.075	-0.12	0.07	0.093	0.202	0.12	0.04	0.009	0.020
Housework 30mins-2hrs (Ref. <30mins)	-0.10	0.03	0.005	0.012	-0.02	0.03	0.427	0.617	0.03	0.01	0.018	0.033
Housework ≥3hrs (Ref. <30mins)	-0.20	0.05	0.000	0.000	-0.01	0.05	0.785	0.816	0.04	0.02	0.132	0.181
Looking after children 30mins-2hrs (Ref. <30mins)	-0.01	0.08	0.888	0.962	0.04	0.08	0.669	0.758	0.05	0.04	0.253	0.329
Looking after children ≥3hrs (Ref. <30mins)	0.08	0.11	0.466	0.577	0.19	0.11	0.080	0.189	0.05	0.06	0.422	0.499
Gardening <30mins (Ref. none)	-0.16	0.03	0.000	0.000	-0.14	0.03	0.000	0.000	0.05	0.02	0.004	0.010
Gardening ≥30mins (Ref. none)	-0.31	0.04	0.000	0.000	-0.23	0.04	0.000	0.000	0.16	0.02	0.000	0.000
Exercising <30mins (Ref. none)	-0.14	0.04	0.000	0.000	0.00	0.04	0.921	0.921	0.09	0.02	0.000	0.000
Exercising ≥30mins (Ref. none)	-0.35	0.04	0.000	0.000	-0.19	0.04	0.000	0.000	0.21	0.02	0.000	0.000
Reading <30mins (Ref. none)	-0.04	0.04	0.317	0.434	-0.02	0.03	0.520	0.676	0.03	0.02	0.120	0.173
Reading ≥30mins (Ref. none)	-0.12	0.05	0.013	0.024	-0.16	0.04	0.000	0.000	0.06	0.02	0.005	0.012
Hobby <30mins (Ref. none)	-0.04	0.03	0.210	0.321	0.01	0.03	0.683	0.758	0.03	0.02	0.054	0.094
Hobby ≥30mins (Ref. none)	-0.17	0.03	0.000	0.000	-0.08	0.03	0.003	0.011	0.11	0.02	0.000	0.000
Communication 30mins-2hrs (Ref. <30mins)	-0.08	0.03	0.008	0.017	0.02	0.03	0.547	0.677	0.04	0.01	0.002	0.006
Communication ≥3hrs (Ref. <30mins)	-0.01	0.05	0.882	0.962	0.11	0.04	0.013	0.042	0.06	0.02	0.010	0.020
COVID-19 news 30mins-2hrs (Ref. <30mins)	0.27	0.03	0.000	0.000	0.45	0.03	0.000	0.000	-0.12	0.01	0.000	0.000
COVID -19 news ≥3hrs (Ref. <30mins)	0.52	0.05	0.000	0.000	0.84	0.04	0.000	0.000	-0.25	0.02	0.000	0.000
Watching TV 30mins-2hrs (Ref. <30mins)	-0.02	0.05	0.644	0.761	-0.04	0.05	0.380	0.581	0.01	0.02	0.680	0.737
Watching TV ≥3hrs (Ref. <30mins)	0.13	0.06	0.029	0.050	0.02	0.06	0.700	0.758	-0.05	0.03	0.063	0.102
Listening radio/music 30mins-2hrs (Ref. <30mins)	-0.09	0.04	0.012	0.024	-0.02	0.03	0.477	0.653	0.03	0.02	0.082	0.125
Listening radio/music ≥3hrs (Ref. <30mins)	-0.25	0.06	0.000	0.000	-0.11	0.05	0.020	0.058	0.08	0.03	0.002	0.006
Internet/social media 30mins-2hrs (Ref. <30mins)	0.02	0.03	0.422	0.549	-0.03	0.03	0.237	0.474	-0.01	0.01	0.723	0.752
Internet/social media ≥3hrs (Ref. <30mins)	0.06	0.05	0.242	0.350	-0.04	0.04	0.363	0.581	0.01	0.02	0.830	0.830
Number of observations	239,005				239,005				239,005			
Number of individuals	41,728				41,728				41,728			

Notes: q values are p values controlling for the positive false discovery rate.

Table S5 Results from the Arellano-Bond models on depression, anxiety and life satisfaction (excluding key workers)

	Model I-ii Depression				Model II-ii Anxiety				Model III-ii Life satisfaction			
	Coef.	SE	p	q†	Coef.	SE	p	q†	Coef.	SE	p	q†
Working 30mins-2hrs (Ref. <30mins)	0.15	0.38	0.685	0.971	0.29	0.35	0.398	0.566	-0.48	0.18	0.007	0.032
Working ≥3hrs (Ref. <30mins)	-0.04	0.33	0.913	0.971	0.96	0.29	0.001	0.005	-0.30	0.15	0.047	0.159
Volunteering <30mins (Ref. none)	-0.05	0.58	0.937	0.971	-0.54	0.61	0.380	0.566	0.15	0.37	0.679	0.833
Volunteering ≥30mins (Ref. none)	-0.03	0.73	0.971	0.971	-0.09	0.53	0.873	0.928	0.34	0.27	0.207	0.373
Housework 30mins-2hrs (Ref. <30mins)	0.03	0.30	0.934	0.971	0.04	0.28	0.894	0.928	-0.05	0.15	0.736	0.839
Housework ≥3hrs (Ref. <30mins)	-0.03	0.49	0.948	0.971	-0.44	0.44	0.308	0.566	0.07	0.25	0.776	0.839
Looking after children 30mins-2hrs (Ref. <30mins)	1.43	0.79	0.069	0.155	1.66	0.80	0.037	0.125	-0.63	0.39	0.108	0.208
Looking after children ≥3hrs (Ref. <30mins)	0.91	0.93	0.332	0.527	2.11	0.95	0.026	0.117	-1.36	0.46	0.003	0.020
Gardening <30mins (Ref. none)	-1.42	0.32	0.000	0.000	-1.02	0.29	0.000	0.000	0.79	0.16	0.000	0.000
Gardening ≥30mins (Ref. none)	-1.43	0.30	0.000	0.000	-0.53	0.27	0.049	0.146	1.10	0.16	0.000	0.000
Exercising <30mins (Ref. none)	-0.41	0.37	0.265	0.477	0.28	0.32	0.380	0.566	0.33	0.19	0.087	0.208
Exercising ≥30mins (Ref. none)	-0.96	0.39	0.015	0.058	0.30	0.33	0.369	0.566	0.49	0.18	0.007	0.032
Reading <30mins (Ref. none)	-0.33	0.38	0.379	0.569	-0.08	0.33	0.812	0.928	0.30	0.19	0.105	0.208
Reading ≥30mins (Ref. none)	-1.06	0.37	0.005	0.023	-0.52	0.33	0.116	0.285	0.46	0.18	0.012	0.046
Hobby <30mins (Ref. none)	1.04	0.35	0.003	0.016	0.67	0.31	0.033	0.125	-0.07	0.16	0.667	0.833
Hobby ≥30mins (Ref. none)	-0.02	0.30	0.938	0.971	0.13	0.29	0.650	0.798	0.09	0.15	0.542	0.732
Communication 30mins-2hrs (Ref. <30mins)	0.32	0.30	0.292	0.493	0.05	0.27	0.844	0.928	0.28	0.15	0.058	0.174
Communication ≥3hrs (Ref. <30mins)	0.62	0.47	0.186	0.359	-0.40	0.41	0.330	0.566	0.20	0.24	0.401	0.583
COVID-19 news 30mins-2hrs (Ref. <30mins)	0.84	0.19	0.000	0.000	1.20	0.17	0.000	0.000	-0.17	0.09	0.069	0.186
COVID -19 news ≥3hrs (Ref. <30mins)	0.70	0.33	0.033	0.099	1.56	0.28	0.000	0.000	-0.26	0.16	0.102	0.208
Watching TV 30mins-2hrs (Ref. <30mins)	-0.08	0.48	0.865	0.971	0.24	0.49	0.633	0.798	-0.01	0.25	0.965	0.965
Watching TV ≥3hrs (Ref. <30mins)	0.02	0.55	0.967	0.971	-0.32	0.54	0.557	0.752	-0.28	0.28	0.325	0.516
Listening radio/music 30mins-2hrs (Ref. <30mins)	-0.72	0.37	0.051	0.138	-0.01	0.32	0.974	0.974	-0.05	0.18	0.777	0.839
Listening radio/music ≥3hrs (Ref. <30mins)	-1.22	0.52	0.020	0.068	0.90	0.47	0.054	0.146	0.20	0.24	0.410	0.583
Internet/social media 30mins-2hrs (Ref. <30mins)	0.53	0.33	0.107	0.222	0.26	0.28	0.351	0.566	-0.04	0.16	0.820	0.852
Internet/social media ≥3hrs (Ref. <30mins)	0.91	0.50	0.066	0.155	0.38	0.45	0.394	0.566	-0.25	0.24	0.295	0.498
First lag of the outcome variable	0.09	0.02	0.000	0.000	0.17	0.02	0.000	0.000	0.09	0.01	0.000	0.000
Number of observations	125,211				125,211				125,211			
Number of individuals	32,855				32,855				32,855			

Notes: q values are p values controlling for the positive false discovery rate.

Table S6 Results from the fixed-effects models on depression, anxiety and life satisfaction (key workers)

	Model I-i Depression				Model II-i Anxiety				Model III-i Life satisfaction			
	Coef.	SE	p	q†	Coef.	SE	p	q†	Coef.	SE	p	q†
Working 30mins-2hrs (Ref. <30mins)	-0.16	0.10	0.106	0.159	0.03	0.08	0.725	0.851	0.01	0.05	0.767	0.928
Working ≥3hrs (Ref. <30mins)	-0.36	0.09	0.000	0.000	0.00	0.06	0.955	0.955	0.14	0.04	0.000	0.000
Volunteering <30mins (Ref. none)	0.01	0.14	0.918	0.918	0.18	0.11	0.104	0.201	-0.15	0.07	0.031	0.070
Volunteering ≥30mins (Ref. none)	0.11	0.19	0.564	0.692	0.02	0.14	0.864	0.897	-0.01	0.09	0.877	0.928
Housework 30mins-2hrs (Ref. <30mins)	-0.19	0.06	0.002	0.006	-0.08	0.06	0.143	0.255	0.08	0.03	0.004	0.012
Housework ≥3hrs (Ref. <30mins)	-0.26	0.10	0.012	0.025	-0.12	0.09	0.199	0.283	0.16	0.05	0.001	0.003
Looking after children 30mins-2hrs (Ref. <30mins)	-0.04	0.13	0.731	0.789	0.15	0.11	0.151	0.255	0.01	0.06	0.872	0.928
Looking after children ≥3hrs (Ref. <30mins)	0.05	0.19	0.807	0.838	0.13	0.16	0.418	0.537	0.06	0.09	0.522	0.742
Gardening <30mins (Ref. none)	-0.11	0.07	0.102	0.159	-0.20	0.06	0.001	0.004	0.09	0.03	0.010	0.027
Gardening ≥30mins (Ref. none)	-0.28	0.08	0.000	0.000	-0.26	0.07	0.000	0.000	0.18	0.04	0.000	0.000
Exercising <30mins (Ref. none)	-0.35	0.09	0.000	0.000	-0.12	0.07	0.080	0.166	0.12	0.04	0.001	0.003
Exercising ≥30mins (Ref. none)	-0.50	0.09	0.000	0.000	-0.35	0.07	0.000	0.000	0.26	0.04	0.000	0.000
Reading <30mins (Ref. none)	-0.19	0.07	0.010	0.023	-0.20	0.07	0.005	0.015	0.03	0.03	0.462	0.734
Reading ≥30mins (Ref. none)	-0.22	0.08	0.008	0.020	-0.29	0.07	0.000	0.000	0.02	0.04	0.690	0.887
Hobby <30mins (Ref. none)	-0.13	0.07	0.070	0.126	-0.07	0.06	0.254	0.343	0.00	0.03	0.973	0.973
Hobby ≥30mins (Ref. none)	-0.18	0.07	0.007	0.019	-0.17	0.06	0.008	0.022	0.02	0.03	0.509	0.742
Communication 30mins-2hrs (Ref. <30mins)	0.02	0.06	0.697	0.784	0.12	0.06	0.051	0.115	0.04	0.03	0.142	0.274
Communication ≥3hrs (Ref. <30mins)	0.04	0.09	0.679	0.784	0.11	0.08	0.170	0.270	0.04	0.04	0.404	0.682
COVID-19 news 30mins-2hrs (Ref. <30mins)	0.39	0.05	0.000	0.000	0.62	0.05	0.000	0.000	-0.24	0.02	0.000	0.000
COVID -19 news ≥3hrs (Ref. <30mins)	0.71	0.12	0.000	0.000	1.09	0.10	0.000	0.000	-0.43	0.05	0.000	0.000
Watching TV 30mins-2hrs (Ref. <30mins)	-0.08	0.10	0.429	0.552	-0.02	0.07	0.763	0.858	0.06	0.04	0.214	0.385
Watching TV ≥3hrs (Ref. <30mins)	0.12	0.11	0.297	0.401	0.02	0.09	0.845	0.897	-0.02	0.06	0.665	0.887
Listening radio/music 30mins-2hrs (Ref. <30mins)	-0.11	0.07	0.094	0.159	-0.13	0.06	0.029	0.071	0.05	0.04	0.139	0.274
Listening radio/music ≥3hrs (Ref. <30mins)	-0.20	0.10	0.041	0.079	-0.05	0.10	0.632	0.776	0.12	0.05	0.025	0.061
Internet/social media 30mins-2hrs (Ref. <30mins)	0.09	0.07	0.174	0.247	0.08	0.06	0.191	0.283	-0.01	0.03	0.821	0.928
Internet/social media ≥3hrs (Ref. <30mins)	0.31	0.10	0.002	0.006	0.27	0.09	0.003	0.010	-0.01	0.04	0.894	0.928
Number of observations	69,177				69,177				69,177			
Number of individuals	12,904				12,904				12,904			

Notes: q values are p values controlling for the positive false discovery rate.

Table S7 Results from the Arellano-Bond models on depression, anxiety and life satisfaction (key workers)

	Model I-ii Depression				Model II-ii Anxiety				Model III-ii Life satisfaction			
	Coef.	SE	p	q†	Coef.	SE	p	q†	Coef.	SE	p	q†
Working 30mins-2hrs (Ref. <30mins)	-0.41	0.46	0.369	0.712	0.61	0.43	0.158	0.427	-0.19	0.21	0.374	0.837
Working ≥3hrs (Ref. <30mins)	0.31	0.29	0.292	0.606	0.42	0.25	0.091	0.351	-0.21	0.13	0.101	0.545
Volunteering <30mins (Ref. none)	0.01	0.81	0.993	0.993	0.10	0.69	0.887	0.993	0.01	0.35	0.988	0.988
Volunteering ≥30mins (Ref. none)	-1.22	0.54	0.024	0.162	0.10	0.67	0.882	0.993	0.74	0.38	0.053	0.358
Housework 30mins-2hrs (Ref. <30mins)	0.38	0.33	0.255	0.574	0.29	0.29	0.317	0.658	0.02	0.15	0.908	0.988
Housework ≥3hrs (Ref. <30mins)	0.32	0.44	0.464	0.783	-0.01	0.45	0.977	0.993	0.06	0.25	0.812	0.988
Looking after children 30mins-2hrs (Ref. <30mins)	0.28	0.53	0.601	0.818	0.46	0.52	0.381	0.735	0.23	0.26	0.372	0.837
Looking after children ≥3hrs (Ref. <30mins)	0.28	0.66	0.671	0.818	1.47	0.72	0.043	0.232	-0.03	0.31	0.924	0.988
Gardening <30mins (Ref. none)	-0.28	0.35	0.421	0.758	-0.50	0.35	0.149	0.427	0.27	0.19	0.144	0.648
Gardening ≥30mins (Ref. none)	-0.54	0.39	0.161	0.435	-0.82	0.36	0.022	0.149	0.38	0.19	0.042	0.358
Exercising <30mins (Ref. none)	-0.05	0.37	0.890	0.961	-0.02	0.35	0.953	0.993	0.20	0.17	0.256	0.768
Exercising ≥30mins (Ref. none)	-0.20	0.38	0.594	0.818	0.23	0.33	0.485	0.818	0.06	0.18	0.735	0.988
Reading <30mins (Ref. none)	0.23	0.46	0.618	0.818	0.30	0.38	0.431	0.776	-0.11	0.19	0.568	0.945
Reading ≥30mins (Ref. none)	0.23	0.46	0.616	0.818	0.64	0.40	0.111	0.375	-0.03	0.21	0.904	0.988
Hobby <30mins (Ref. none)	-0.58	0.35	0.097	0.306	-0.35	0.31	0.251	0.565	-0.14	0.17	0.403	0.837
Hobby ≥30mins (Ref. none)	0.16	0.36	0.664	0.818	0.07	0.30	0.828	0.993	-0.12	0.16	0.469	0.844
Communication 30mins-2hrs (Ref. <30mins)	0.47	0.29	0.102	0.306	-0.05	0.27	0.844	0.993	0.07	0.16	0.655	0.983
Communication ≥3hrs (Ref. <30mins)	-0.04	0.41	0.929	0.965	-0.12	0.40	0.766	0.993	0.27	0.23	0.232	0.768
COVID-19 news 30mins-2hrs (Ref. <30mins)	0.73	0.26	0.005	0.068	1.19	0.27	0.000	0.000	-0.53	0.12	0.000	0.000
COVID -19 news ≥3hrs (Ref. <30mins)	1.85	0.46	0.000	0.000	1.75	0.41	0.000	0.000	-0.91	0.20	0.000	0.000
Watching TV 30mins-2hrs (Ref. <30mins)	-0.19	0.49	0.697	0.818	-0.10	0.41	0.802	0.993	-0.17	0.22	0.441	0.844
Watching TV ≥3hrs (Ref. <30mins)	0.11	0.58	0.847	0.953	0.15	0.51	0.765	0.993	-0.10	0.26	0.713	0.988
Listening radio/music 30mins-2hrs (Ref. <30mins)	-0.54	0.32	0.093	0.306	-0.56	0.30	0.061	0.275	-0.19	0.20	0.339	0.837
Listening radio/music ≥3hrs (Ref. <30mins)	-0.88	0.51	0.083	0.306	-0.57	0.46	0.217	0.533	-0.33	0.26	0.210	0.768
Internet/social media 30mins-2hrs (Ref. <30mins)	0.44	0.37	0.236	0.574	0.00	0.36	0.993	0.993	0.09	0.17	0.595	0.945
Internet/social media ≥3hrs (Ref. <30mins)	0.99	0.50	0.048	0.259	0.12	0.44	0.780	0.993	-0.01	0.25	0.955	0.988
First lag of the outcome variable	0.07	0.03	0.015	0.135	0.14	0.03	0.000	0.000	0.01	0.02	0.772	0.988
Number of observations	34,259				34,259				34,259			
Number of individuals	9,545				9,545				9,545			

Notes: q values are p values controlling for the positive false discovery rate.

Table S8 Results from the fixed-effects models on depression (controlling for anxiety) and anxiety (controlling for depression)

	Model I-i Depression				Model II-i Anxiety			
	Coef.	SE	p	q†	Coef.	SE	p	q†
Working 30mins-2hrs (Ref. <30mins)	-0.05	0.04	0.178	0.253	0.05	0.03	0.123	0.255
Working ≥3hrs (Ref. <30mins)	-0.31	0.03	0.000	0.000	0.19	0.03	0.000	0.000
Volunteering <30mins (Ref. none)	0.01	0.06	0.851	0.851	-0.01	0.05	0.769	0.799
Volunteering ≥30mins (Ref. none)	-0.11	0.08	0.162	0.243	-0.01	0.04	0.754	0.799
Housework 30mins-2hrs (Ref. <30mins)	-0.09	0.02	0.000	0.000	0.02	0.02	0.392	0.504
Housework ≥3hrs (Ref. <30mins)	-0.19	0.04	0.000	0.000	0.06	0.03	0.061	0.137
Looking after children 30mins-2hrs (Ref. <30mins)	-0.07	0.06	0.272	0.350	0.08	0.06	0.142	0.274
Looking after children ≥3hrs (Ref. <30mins)	-0.03	0.08	0.746	0.775	0.14	0.07	0.057	0.137
Gardening <30mins (Ref. none)	-0.06	0.03	0.018	0.035	-0.08	0.02	0.000	0.000
Gardening ≥30mins (Ref. none)	-0.17	0.03	0.000	0.000	-0.09	0.03	0.001	0.003
Exercising <30mins (Ref. none)	-0.17	0.03	0.000	0.000	0.06	0.03	0.018	0.049
Exercising ≥30mins (Ref. none)	-0.26	0.03	0.000	0.000	-0.04	0.03	0.177	0.309
Reading <30mins (Ref. none)	-0.04	0.03	0.231	0.312	-0.02	0.03	0.341	0.485
Reading ≥30mins (Ref. none)	-0.03	0.03	0.403	0.473	-0.12	0.03	0.000	0.000
Hobby <30mins (Ref. none)	-0.06	0.03	0.030	0.054	0.02	0.02	0.288	0.432
Hobby ≥30mins (Ref. none)	-0.11	0.02	0.000	0.000	-0.02	0.02	0.450	0.552
Communication 30mins-2hrs (Ref. <30mins)	-0.08	0.02	0.001	0.003	0.06	0.02	0.003	0.009
Communication ≥3hrs (Ref. <30mins)	-0.06	0.04	0.091	0.145	0.11	0.03	0.001	0.003
COVID-19 news 30mins-2hrs (Ref. <30mins)	0.01	0.02	0.579	0.651	0.34	0.02	0.000	0.000
COVID -19 news ≥3hrs (Ref. <30mins)	0.04	0.04	0.302	0.371	0.62	0.03	0.000	0.000
Watching TV 30mins-2hrs (Ref. <30mins)	-0.01	0.03	0.684	0.739	-0.02	0.03	0.576	0.648
Watching TV ≥3hrs (Ref. <30mins)	0.12	0.04	0.006	0.015	-0.04	0.04	0.285	0.432
Listening radio/music 30mins-2hrs (Ref. <30mins)	-0.07	0.03	0.012	0.025	0.00	0.02	0.953	0.953
Listening radio/music ≥3hrs (Ref. <30mins)	-0.18	0.04	0.000	0.000	0.02	0.04	0.503	0.590
Internet/social media 30mins-2hrs (Ref. <30mins)	0.04	0.02	0.052	0.088	-0.03	0.02	0.183	0.309
Internet/social media ≥3hrs (Ref. <30mins)	0.09	0.04	0.010	0.023	-0.03	0.03	0.368	0.497
Depression/anxiety	0.58	0.01	0.000	0.000	0.49	0.00	0.000	0.000
Number of observations	239,005				239,005			
Number of individuals	41,728				41,728			

Notes: q values are p values controlling for the positive false discovery rate.

Table S9 Results from the fixed-effects models on depression, anxiety and life satisfaction (restricting to participants with at least three repeated measures)

	Model I-i Depression				Model II-i Anxiety				Model III-i Life satisfaction			
	Coef.	SE	p	q†	Coef.	SE	p	q†	Coef.	SE	p	q†
Working 30mins-2hrs (Ref. <30mins)	-0.04	0.04	0.334	0.410	0.04	0.04	0.308	0.438	0.02	0.02	0.322	0.395
Working ≥3hrs (Ref. <30mins)	-0.27	0.04	0.000	0.000	0.06	0.04	0.109	0.210	0.11	0.02	0.000	0.000
Volunteering <30mins (Ref. none)	0.00	0.06	0.956	0.956	-0.02	0.05	0.695	0.722	-0.01	0.03	0.747	0.835
Volunteering ≥30mins (Ref. none)	-0.16	0.10	0.098	0.139	-0.10	0.06	0.140	0.252	0.06	0.04	0.137	0.195
Housework 30mins-2hrs (Ref. <30mins)	-0.10	0.03	0.000	0.000	-0.03	0.03	0.242	0.384	0.05	0.01	0.000	0.000
Housework ≥3hrs (Ref. <30mins)	-0.19	0.05	0.000	0.000	-0.03	0.04	0.459	0.574	0.06	0.02	0.004	0.008
Looking after children 30mins-2hrs (Ref. <30mins)	-0.02	0.07	0.820	0.858	0.08	0.07	0.226	0.381	0.01	0.03	0.744	0.835
Looking after children ≥3hrs (Ref. <30mins)	0.04	0.10	0.646	0.727	0.16	0.09	0.083	0.172	0.01	0.04	0.841	0.850
Gardening <30mins (Ref. none)	-0.15	0.03	0.000	0.000	-0.15	0.03	0.000	0.000	0.05	0.02	0.001	0.002
Gardening ≥30mins (Ref. none)	-0.32	0.03	0.000	0.000	-0.24	0.03	0.000	0.000	0.16	0.02	0.000	0.000
Exercising <30mins (Ref. none)	-0.19	0.04	0.000	0.000	-0.02	0.03	0.587	0.689	0.10	0.02	0.000	0.000
Exercising ≥30mins (Ref. none)	-0.39	0.04	0.000	0.000	-0.24	0.03	0.000	0.000	0.22	0.02	0.000	0.000
Reading <30mins (Ref. none)	-0.07	0.03	0.046	0.069	-0.06	0.03	0.055	0.135	0.03	0.02	0.107	0.170
Reading ≥30mins (Ref. none)	-0.13	0.04	0.001	0.002	-0.17	0.04	0.000	0.000	0.05	0.02	0.006	0.011
Hobby <30mins (Ref. none)	-0.05	0.03	0.130	0.167	0.00	0.03	0.964	0.964	0.02	0.01	0.162	0.219
Hobby ≥30mins (Ref. none)	-0.18	0.03	0.000	0.000	-0.10	0.03	0.000	0.000	0.09	0.01	0.000	0.000
Communication 30mins-2hrs (Ref. <30mins)	-0.07	0.03	0.009	0.015	0.03	0.02	0.257	0.386	0.04	0.01	0.001	0.002
Communication ≥3hrs (Ref. <30mins)	-0.01	0.04	0.826	0.858	0.10	0.04	0.005	0.015	0.06	0.02	0.005	0.010
COVID-19 news 30mins-2hrs (Ref. <30mins)	0.30	0.02	0.000	0.000	0.48	0.02	0.000	0.000	-0.14	0.01	0.000	0.000
COVID -19 news ≥3hrs (Ref. <30mins)	0.59	0.05	0.000	0.000	0.90	0.04	0.000	0.000	-0.29	0.02	0.000	0.000
Watching TV 30mins-2hrs (Ref. <30mins)	-0.03	0.04	0.428	0.502	-0.03	0.04	0.421	0.568	0.02	0.02	0.221	0.284
Watching TV ≥3hrs (Ref. <30mins)	0.14	0.05	0.010	0.016	0.04	0.05	0.468	0.574	-0.04	0.02	0.114	0.171
Listening radio/music 30mins-2hrs (Ref. <30mins)	-0.09	0.03	0.002	0.004	-0.05	0.03	0.062	0.140	0.03	0.02	0.038	0.064
Listening radio/music ≥3hrs (Ref. <30mins)	-0.24	0.05	0.000	0.000	-0.11	0.04	0.010	0.027	0.09	0.02	0.000	0.000
Internet/social media 30mins-2hrs (Ref. <30mins)	0.04	0.03	0.104	0.140	-0.01	0.02	0.668	0.721	0.00	0.01	0.850	0.850
Internet/social media ≥3hrs (Ref. <30mins)	0.12	0.05	0.008	0.014	0.02	0.04	0.618	0.695	0.01	0.02	0.773	0.835
Number of observations	239,005				239,005				239,005			
Number of individuals	41,728				41,728				41,728			

Notes: q values are p values controlling for the positive false discovery rate.
